# Supplementary material for: An open-source framework for large-scale, flexible evaluation of biomedical text mining systems
Source: J Biomed Discov Collab. 2008 Jan 29;3:1. doi: 10.1186/1747-5333-3-1 (PMC2276192; doi:10.1186/1747-5333-3-1)
Supplement: Additional file 1 — Evaluation platform source code. The additional file contains the software evaluation framework discussed in this paper. The most current release can be downloaded from SourceForge.net [1]. See the accompanying README file for instructions on installation and use. Also included in the distribution is a collection of UIMA wrappers for some commonly used BioNLP tools and annotated corpora. [file 1747-5333-3-1-S1.zip › bionlp_uima_repository-1.0.1/docs/api/edu/uchsc/ccp/uima/mention/CCPClassMention_Type.html]

CCPClassMention\_Type (Center for Computational Pharmacology Util/UIMA API)


|  |  |  |
| --- | --- | --- |
| **Overview** \| **Package** \| **Class** \| **Tree** \| **Deprecated** \| **Index** \| **Help** | |  |
| **PREV CLASS**   **NEXT CLASS** | **FRAMES**    **NO FRAMES**     **All Classes** |  |
| SUMMARY: NESTED \| FIELD \| CONSTR \| METHOD | DETAIL: FIELD \| CONSTR \| METHOD |  |


---


## edu.uchsc.ccp.uima.mention Class CCPClassMention\_Type

```
java.lang.Object
  org.apache.uima.jcas.cas.TOP_Type
      edu.uchsc.ccp.uima.mention.CCPMention_Type
          edu.uchsc.ccp.uima.mention.CCPClassMention_Type
```

---

``` public class CCPClassMention_Type extends CCPMention_Type ```

The CCP ClassMention is the root of a flexible class structure that can be used to store virtually any frame-based representation of a particular class. Common class mention types include, but are not limited to, such things as entities (protein, cell type, cell line, disease, tissue, etc.) and frames (interaction, transport, regulation, etc.).
Updated by JCasGen Tue Aug 28 16:44:10 MDT 2007

---

| **Field Summary** | |
| --- | --- |
| `static boolean` | `featOkTst` |
| `static int` | `typeIndexID` |

**Fields inherited from class org.apache.uima.jcas.cas.TOP\_Type**

`casImpl, casType, casTypeCode, instanceOf_Type, jcas, ll_cas, lowLevelArrayBoundChecks, lowLevelTypeChecks, useExistingInstance`


**Constructor Summary**

`CCPClassMention_Type(org.apache.uima.jcas.JCas jcas,
org.apache.uima.cas.Type casType)`
  
          initialize variables to correspond with Cas Type and Features


| **Method Summary** | |
| --- | --- |
| `int` | `getCcpTextAnnotations(int addr)` |
| `int` | `getCcpTextAnnotations(int addr, int i)` |
| `protected  org.apache.uima.cas.impl.FSGenerator` | `getFSGenerator()` |
| `int` | `getSlotMentions(int addr)` |
| `int` | `getSlotMentions(int addr, int i)` |
| `void` | `setCcpTextAnnotations(int addr, int v)` |
| `void` | `setCcpTextAnnotations(int addr, int i, int v)` |
| `void` | `setSlotMentions(int addr, int v)` |
| `void` | `setSlotMentions(int addr, int i, int v)` |

**Methods inherited from class edu.uchsc.ccp.uima.mention.CCPMention\_Type**

`getMentionName, setMentionName`

**Methods inherited from class org.apache.uima.jcas.cas.TOP\_Type**

`addToIndexes, checkType, getTypeIndexID, invalidTypeArg, noObjCreate, removeFromIndexes`

**Methods inherited from class java.lang.Object**

`clone, equals, finalize, getClass, hashCode, notify, notifyAll, toString, wait, wait, wait`

**Field Detail**

### typeIndexID

```
public static final int typeIndexID
```

---


### featOkTst

```
public static final boolean featOkTst
```


**Constructor Detail**

### CCPClassMention\_Type

```
public CCPClassMention_Type(org.apache.uima.jcas.JCas jcas,
                            org.apache.uima.cas.Type casType)
```

:   initialize variables to correspond with Cas Type and Features


**Method Detail**

### getFSGenerator

```
protected org.apache.uima.cas.impl.FSGenerator getFSGenerator()
```

:   **Overrides:**: `getFSGenerator` in class `CCPMention_Type`

---


### getSlotMentions

```
public int getSlotMentions(int addr)
```

---


### setSlotMentions

```
public void setSlotMentions(int addr,
                            int v)
```

---


### getSlotMentions

```
public int getSlotMentions(int addr,
                           int i)
```

---


### setSlotMentions

```
public void setSlotMentions(int addr,
                            int i,
                            int v)
```

---


### getCcpTextAnnotations

```
public int getCcpTextAnnotations(int addr)
```

---


### setCcpTextAnnotations

```
public void setCcpTextAnnotations(int addr,
                                  int v)
```

---


### getCcpTextAnnotations

```
public int getCcpTextAnnotations(int addr,
                                 int i)
```

---


### setCcpTextAnnotations

```
public void setCcpTextAnnotations(int addr,
                                  int i,
                                  int v)
```


---


|  |  |  |
| --- | --- | --- |
| **Overview** \| **Package** \| **Class** \| **Tree** \| **Deprecated** \| **Index** \| **Help** | |  |
| **PREV CLASS**   **NEXT CLASS** | **FRAMES**    **NO FRAMES**     **All Classes** |  |
| SUMMARY: NESTED \| FIELD \| CONSTR \| METHOD | DETAIL: FIELD \| CONSTR \| METHOD |  |


---
